# AMPK and CAMKK activation participate in early events of *Toxoplasma gondii*-triggered NET formation in bovine polymorphonuclear neutrophils

**DOI:** 10.3389/fvets.2025.1557509

**Published:** 2025-03-18

**Authors:** Iván Conejeros, Zahady D. Velásquez, Gabriel Espinosa, Lisbeth Rojas-Baron, Magdalena Grabbe, Carlos Hermosilla, Anja Taubert

**Affiliations:** Institute of Parasitology, Justus Liebig University Giessen, Giessen, Germany

**Keywords:** PMN, *Toxoplasma gondii*, cattle, NET, AMPK, CAMKK, innate immunity, bovine

## Abstract

*Toxoplasma gondii* is an obligate intracellular apicomplexan parasite that infects humans, eventually causing severe diseases like prenatal or ocular toxoplasmosis. *T. gondii* also infects cattle but rarely induces clinical signs in this intermediate host type. So far, the innate immune mechanisms behind the potential resistance of bovines to clinical *T. gondii* infections remain unclear. Here, we present evidence on sustained activation of bovine polymorphonuclear neutrophils PMN by *T. gondii* tachyzoites, which is linked to a rise in cytoplasmic calcium concentrations, an enhancement of calcium/calmodulin-dependent protein kinase kinase 2 (CAMKK) and AMP-activated protein kinase (AMPK). NETosis is a specific form of programmed cell death, characterized by the release chromatin from the nucleus to the extracellular space resulting in formation of neutrophil extracellular traps (NETs). NETs can kill and entrap pathogens. In our experiments, NETosis was triggered by *T. gondii*, and this effector mechanism was enhanced by pre-treatments with the AMPK activator AICAR. Moreover, tachyzoite-mediated bovine neutrophil DNA release depended on MAPK- and store operated calcium entry- (SOCE) pathways since it was diminished by the inhibitors UO126 and 2-APB, respectively. Overall, we here provide new insights into early polymorphonuclear neutrophils responses against *T. gondii* for the bovine system.

## 1 Introduction

*Toxoplasma gondii* is a protozoan apicomplexan parasite able to infect virtually all warm-blooded animals including humans. The definitive host of *T. gondii* are felids and it is estimated that at least one third of humanity is currently infected with *T. gondii*. However, *T. gondii* prevalence varies greatly amongst populations and it is influenced by ethnocultural habits (1, 2). One transmission route to humans is the consumption of raw and/or undercooked meat derived from livestock infected with *T. gondii* cysts. Virtually all edible portions of an animal can contain viable *T. gondii* tissue cysts that can survive in food animals for years (2). *T. gondii* infection in naturally infected cattle normally is free of symptoms, suggesting that cattle are resistant to clinical toxoplasmosis. The seroprevalence of toxoplasmosis in cattle is unclear, varies greatly from region to region and is highly dependent on the type of the bioassay (3).

One of the causes of the hypothesized resistance of cattle to clinical *T. gondii* infection is the innate immune response. Among the cells of the innate immune system, polymorphonuclear neutrophils (PMN) form a first line of cellular defence against pathogens (4). PMN are direct immune effector cells, and own at least three effector mechanisms: phagocytosis, degranulation and neutrophil extracellular trap (NET) formation. In addition, PMN can produce reactive oxygen species (ROS), which may directly kill microorganisms, and release cytokines, which modulate other innate and also adaptative immune responses. For example, PMN-derived factors enhance dendritic cell (DC) recruitment and antigen presentation, T cellular cytokine production and B cell expansion (5).

PMN are capable to produce and release NETs, denominated as NETosis or NETotic process. First described as a suicidal mechanism to extracellularly kill pathogens (6), NETs are composed of chromatin fibres which contain enzymes mainly present in azurophilic granules, such as neutrophil elastase (NE), cathelicidin (LL-37) and myeloperoxidase (MPO) (7, 8). The DNA backbone of NETs mainly originates from the multilobulated nuclei but can also contain mitochondrial DNA (9). Initially described as an antimicrobial mechanism, activators of NET formation were expanded to viruses (10), fungi (11), crystals (12) and parasites (13). Among parasites, protozoan apicomplexa like *Neospora caninum* (14), *Benoitia besnoiti* (15, 16), *Eimeria bovis* (17, 18) and *Cryptosporidium parvum* (19) – among others – were shown to induce NETs in different animal species, thereby indicating the conserved nature of NET formation as antiparasitic defence mechanism. *T. gondii* stages were also reported to induce NETosis in humans (20), mice (20, 21), cattle (22, 23), sheep (22), donkeys (24), dolphins (*Tursiops truncatus*) (25), harbour seals (*Phoca vitulina*) (26) and in definitive hosts of *T. gondii*, domestic cats (27, 28).

Several early events (≤ 30 min) of the multistep process of NETosis were identified. Amongst others, an increased ROS production seems necessary with the consequent stimulation of MPO that later mediates NE translocation from PMN granules to the nucleus (8, 29). Also, the activation of protein-arginine deaminase 4 (PAD4), which later will induce chromatin decondensation and nuclear membrane disruption (8), was identified as pivotal factor of NET formation. One important chemical mediator of NET formation is calcium. A rise in intracellular calcium concentration ([Ca^2+^]_i_) was shown to be involved in LPS- but not in *Candida albicans*-induced NETosis (30). In line, chelation of extracellular calcium led to impaired NETosis (8). Moreover, treatments with calcium ionophores like A23187 and ionomycin caused NET release (30) and PAD4 activation depended on a high calcium concentration (31, 32). Consequently, an increase in intracellular calcium, either via the release from intracellular stores or by influx from the extracellular space, is key for NETosis. Thus, the identification of cellular targets of calcium during NETosis will contribute to the knowledge on molecular mechanisms of PMN activation and NET release (32). Downstream of a [Ca^2+^]_i_ rise, several kinases were demonstrated to be involved in NET formation like Akt (33), Raf–MEK–ERK pathway (34) and phospho-inositide 3-kinase (PI3K) (35). For bovine PMN, chemical inhibition experiments have shown that calcium signalling and downstream activation of molecular pathways like calcium/calmodulin-dependent protein kinase kinase (CAMKK), AMP-activated kinase (AMPK) and MAPKK pathways play a pivotal role in *E. bovis-* and *B. besnoiti-*induced PMN activation (36–38). AMPK activation is key to PMN chemotaxis and bacterial killing and also counteracts the inhibition of chemotaxis induced by LPS (at concentrations higher than 30 ng/mL) (39, 40). Chemical inhibition of AMPK in human PMN reduced fMLP and PMA-induced ROS production (41). AMPK also regulates autophagy since it directly activates the autophagic pre-initiation complex ULK-1 through phosphorylation (42). Interestingly, autophagy and NET formation seem intrinsically linked and a concomitant occurrence is observed in parasite- (37, 38) and PMA-stimulated PMN (43–45).

In the current work we provide new evidence on the involvement of an intracellular calcium concentration rise, CAMKK and AMPK activation, modulation of autophagic proteins like ULK-1 and Beclin-1 and several canonical signalling pathways like PI3K and store-operated calcium entry (SOCE) in *T. gondii*-induced NETosis of bovine PMN.

## 2 Materials and methods

### 2.1 Ethics statement

The current study was performed in accordance with the Justus Liebig University Giessen Animal Care Committee Guidelines. Protocols were approved by the Ethics Commission for Experimental Animal Studies of the Federal State of Hesse (Regierungspräsidium Giessen; GI 18/10 Nr. V 2/2022; JLU-No. 0002_V) and are in accordance with European Animal Welfare Legislation: ART13TFEU, and currently applicable German Animal Protection Laws.

### 2.2 Bovine PMN isolation

Peripheral blood was collected from six different animals in heparinized sterile plastic tubes (Kabe Labortechnik, Nümbrecht, Germany) from the jugular vein of healthy adult dairy cows. Then, 20 mL of heparinized blood was mixed with 20 mL of sterile PBS containing 0.02% EDTA (Carl Roth, Karlsruhe, Germany), carefully layered on top of 12 mL of Histopaque-1077 separation solution (density = 1.077 g/L; Cat#10771, Sigma-Aldrich, UK) and centrifuged [800× g, 45 min, room temperature (RT)] without brake. After removal of plasma and the buffy coat containing peripheral blood mononuclear cells, the cell pellet was suspended in 20 mL of lysis buffer (5.5 mM NaH_2_PO_4_, 10.8 mM KH_2_PO_4_; pH 7.2) and gently mixed for 60 s to lyse erythrocytes. Osmolarity was rapidly restored by the addition of 10 mL of hypertonic buffer (462 mM NaCl, 5.5 mM NaH_2_PO_4_, 10.8 mM KH_2_PO_4_; pH 7.2) and 10 mL of Hank’s balanced salt solution (HBSS, 14065–049, Gibco, Paisley, UK). The lysis step was repeated at least twice, increasing the number of erythrocyte-lysis step repetitions until no visible erythrocytes were present on the samples. PMN were then suspended in 5 mL of HBSS, counted in a Neubauer chamber, and allowed to rest on ice for 30 min before any experimental use.

### 2.3 *Toxoplasma gondii* tachyzoite maintenance

*Toxoplasma gondii* (RH strain) tachyzoites were maintained by serial passages in human foreskin fibroblasts (HFF). Therefore, *T. gondii* tachyzoites were obtained by scrapping the *T. gondii*-infected HFF monolayer. Then the parasite- and HFF-containing solution was passed through a syringe with a 25G needle to release *T. gondii* from the host cells. Then, the solution containing harvested *T. gondii* was filtered through a 3 μm filter and centrifuged at 3000 × g for 10 min. The parasite pellet was suspended in RPMI medium, and used for bovine PMN confrontation after the determination of the concentration in a Neubauer chamber. All experiments were performed at a ratio of 1:4 (PMN: *T. gondii*).

### 2.4 Flow cytometry-based measurements of PMN intracellular calcium concentration

PMN were adjusted to a concentration of 1 × 10^7^ PMN/mL and incubated with 1 μM FLUO-4 AM (Invitrogen, UK) for 30 min at 37°C and 5% CO_2_. Then FLUO-4-loaded PMN were washed twice in HBSS and suspended again at a concentration of 1 × 10^7^ PMN/mL. For each assay, 5 × 10^5^ PMN (50 μL of PMN suspension) were dispensed in flow cytometry tubes adjusting the volume to 400 μL with HBSS. After recording a baseline for 15 s, PMN were stimulated with *T. gondii* (1:4 PMN: tachyzoite ratio), or with the calcium ionophore A23187 (25 μM, Sigma, Germany) thereby immediately registering changes in fluorescence intensity in the FL-1 channel. All calcium influx related experiments were performed in a BD Accuri C6 plus flow cytometer (BD Biosciences, Heidelberg, Germany) equipped with a non-pressurized peristaltic pump, allowing to add *T. gondii* without interrupting sampling. After experimentation, the mean intensity of fluorescence (MFI) after 5 min of stimulation was determined and graphed.

### 2.5 Protein extraction and Western blotting (WB)

PMN-*T. gondii* interactions were performed in 1 mL of RPMI media without phenol red (Sigma Aldrich Cat#R7509, Great Britain) in 1.5 mL-Eppendorf tubes (Greiner Bio-One Cat#682201, Frickenhausen, Germany). For protein extraction, 5 × 10^6^ PMN were confronted with 20 × 10^6^ *T. gondii* tachyzoites (1:4 PMN:parasite ratio). After the desired incubation time, proteins from *T. gondii*-exposed and non-exposed bovine PMN (*n* = 6) were extracted in RIPA buffer (50 mM Tris–HCl, pH 7.4; 1% NP-40; 0.5% Na-deoxycholate; 0.1% SDS; 150 mM NaCl; 2 mM EDTA; 50 mM NaF; all Roth, Karlsruhe, Germany) supplemented with a protease inhibitor cocktail (Sigma-Aldrich). Then, the cell pellet was lysed using an ultrasound sonicator (20 s, 5 cycles). Thereafter, the samples were centrifuged (10,000× g, 10 min, 4°C) to sediment remnant intact cells. The supernatants were collected, and their protein content was quantified via the Pierce™ Bradford Plus Protein Assay Kit (Cat#23236, Thermo Scientific, Rockford, IL, USA) according to the manufacturer’s instructions. For immunoblotting, samples were supplemented with 6 M urea. After boiling (95°C, 5 min), total protein (40 μg per slot) was electrophoresed in 12% or 15% polyacrylamide gels (100 V, 90 min) using a Mini-PROTEAN Tetra Cell system (Biorad, Feldkirchen, Germany). Proteins were then transferred (300 mA, 2 h) to polyvinylidene difluoride (PVDF) membranes (Millipore, Darmstadt, Germany) using a semidry blotting instrument (Mini-transfer blot, Biorad, Feldkirchen, Germany). The blots were first incubated in blocking solution (3% BSA in TBS containing 0.1% Tween, all Sigma-Aldrich; 1 h, RT) and then reacted overnight at 4°C with primary antibodies [anti-AMPKα (Cat#50081 1:1000, Cell Signaling, Leiden, The Netherlands), anti-CAMKK (Cat#ab96531, 1:1000 Abcam, Cambridge, UK), anti-pCAMKK (Cat#abPA5-64569, 1:1000 Thermo Fischer), anti-Beclin-1 (Cat#3495, 1:1000 Cell Signaling, Leiden, The Netherlands), anti-p-Beclin-1 (Cat#14717, 1:1000 Cell Signaling, Leiden, The Netherlands), and anti-ULK1 (Cat#8054, 1:1000 Cell Signaling, Leiden, The Netherlands)] diluted in blocking solution. The detection of vinculin (Cat#sc-73614, 1:1000, Santa Cruz, Texas, LA, USA) was used for normalization of the samples. Signal detection was accomplished by incubation for 30 min at RT in the corresponding secondary antibodies conjugated with peroxidase (Cat#31430, 1:40,000 and Cat#31460, 1:40,000, both Pierce) and then applying an enhanced chemiluminescence detection system (ECL^®^ plus kit, RPN2132, GE Healthcare, Buckinghamshire, UK). Protein signals were recorded in a ChemoCam Imager (Biorad, Feldkirchen, Germany). Protein masses were controlled by a protein ladder (PageRulerplus pre-stained protein ladder covering ~10–250 kDa; Thermo Fisher Scientific, Rockford, IL, USA). Quantification of protein band intensities was performed by Image J software (Fiji version using the gel analyzer plugin).

### 2.6 Immunofluorescence-based detection and quantification of *Toxoplasma gondii*-induced NET formation

Unstimulated bovine PMN (negative control) and PMN confronted with *T. gondii* (1:4 PMN: tachyzoite ratio) were incubated (37°C, 5% CO_2_) on 15 mm glass coverslips pre-coated with 0.01% poly-L-lysine in 6-well cell culture plates (Greiner Bio-One) in a final volume of 1 mL RPMI media without phenol red (Sigma Aldrich R7509, Great Britain) in the presence or absence of 1 mM of AICAR. After 4 h of co-culture, the samples were fixed with 4% paraformaldehyde (15 min, RT). After the fixation step, the samples were carefully washed thrice with sterile PBS and incubated in blocking/permeabilization solution (PBS containing 3% BSA, 0.3% Triton X-100; Sigma-Aldrich, St. Louis, MI, USA) for 1 h at RT. Then, samples were incubated in primary antibodies (anti-histone-H1-DNA, 1:200 Cat# MAB3864, Merck-Millipore, Darmstadt, Germany; anti-neutrophil elastase, NE, Cat# ab6872, Abcam, Cambridge, UK) diluted in blocking/permeabilization solution (overnight, 4°C, humidified chamber). Then, the samples were washed thrice in sterile PBS and incubated for 30 min at RT, protected from light, with corresponding secondary antibodies (anti-rabbit IgG Alexa 488, 1:500, Cat# A11008, and anti-mouse IgG Alexa 594, Cat# A11005, both Thermo Fisher, Eugene, ON, USA). Samples were mounted in glass microscopy slides and the DNA counterstaining was accomplished by 4′,6-diamidin-2-phenylindol (DAPI) present in mounting medium (Fluoromount G, 00–4,959-52, Thermo Fisher, Waltham, MA, USA). Images were acquired by a Nikon Eclipse Ti2-A inverted microscope equipped with a ReScan confocal microscopic instrumentation (RCM 1.1 Visible, Confocal.nl) and a motorized z-stage (DI1500). Three channels were recorded for signal detection: DAPI/Blue/405-laser, AlexaFluor488/Green/Argon-488-laser, and AlexaFluor594/Red/HeNe-543-laser. Images were acquired by a sCMOS camera (PCO edge) using a CFI Plan Apochromat 60 X lambda-immersion oil objective (NA 1.4/0.13; Nikon), controlled by NIS-Elements v 5.11 (Nikon, Tokyo, Japan) software. Samples were imaged via z-stack optical series with a step size of 0.2–0.3 microns in depth. The z-series were displayed as maximum z-projections, and all settings (gamma, brightness, and contrast) were applied at identical conditions when comparing image sets using Image J software, Fiji version (46). Measurements of defined parameters (e.g., area, integrated density and numbers) were performed with Fiji/Image J software (version: 1.53c). Histone H1-DNA and DAPI signals were acquired at the same time point for each image. A manual threshold was applied to each channel using the clustering algorithm of Otsu (47). Sharpness of the images was adjusted and the percentage of cells releasing NETs for each experimental condition was assessed, as described by Brinkmann et al. (48) by determining the number of cells positive in the DNA-H1 channel and the total number of cells in the DAPI channel. Cells positive for DNA-H1 and with an expanded non-multilobulated nucleus were defined as NETotic.

### 2.7 Quantification of extracellular DNA release

Using 96-well flat bottom plates (Greiner Bio-One, Germany), 2 × 10^5^ bovine PMN were cocultured with *T. gondii* (1:4 PMN: tachyzoite ratio) in RPMI 1640 medium at a final volume of 200 μL. PMN only and plain media samples were used as negative controls. For chemical inhibition experiments, PMN were pretreated with the MAPK inhibitor UO126 (50 μM, 30 min; Sigma-Aldrich), the PI3K inhibitor LY294002 (1 μM, 30 min; CST) or the SOCE inhibitor 2-APB (50 μM, 30 min; Sigma) before exposure to *T. gondii*. To prove that the source of the fluorescence signal is extruded DNA from PMN, a well containing DNase I (90 U/sample, Roche Diagnostics) was included in the experimental setting. DNA was quantified via PicoGreen-based dsDNA quantitation reagent (5 μM, Invitrogen)-derived fluorescence intensities being assessed at an excitation wavelength of 500 nm and emission wavelength 525 nm using an automated plate monochrome reader (Varioskan Flash; Thermo Scientific) after 4 h of co-culture. Results are expressed in relative fluorescence units (RFU).

### 2.8 Statistical analysis

Statistical significance was defined by a *p*-value <0.05. In Western blot experiments, the *p*-values were calculated via paired, two-tailed *t*-tests, comparing control PMN vs. PMN incubated with *T. gondii*. ANOVA followed by Dunnet multiple comparisons tests were applied to calcium influx and DNA release data. Bar graphs represent the mean ± SD, and statistical analysis was performed by GraphPad software (v. 7.03).

## 3 Results

### 3.1 Exposure to *Toxoplasma gondii* induces a rise in intracellular calcium concentration [Ca^2+^]_i_ in bovine PMN

**Figure 1:**
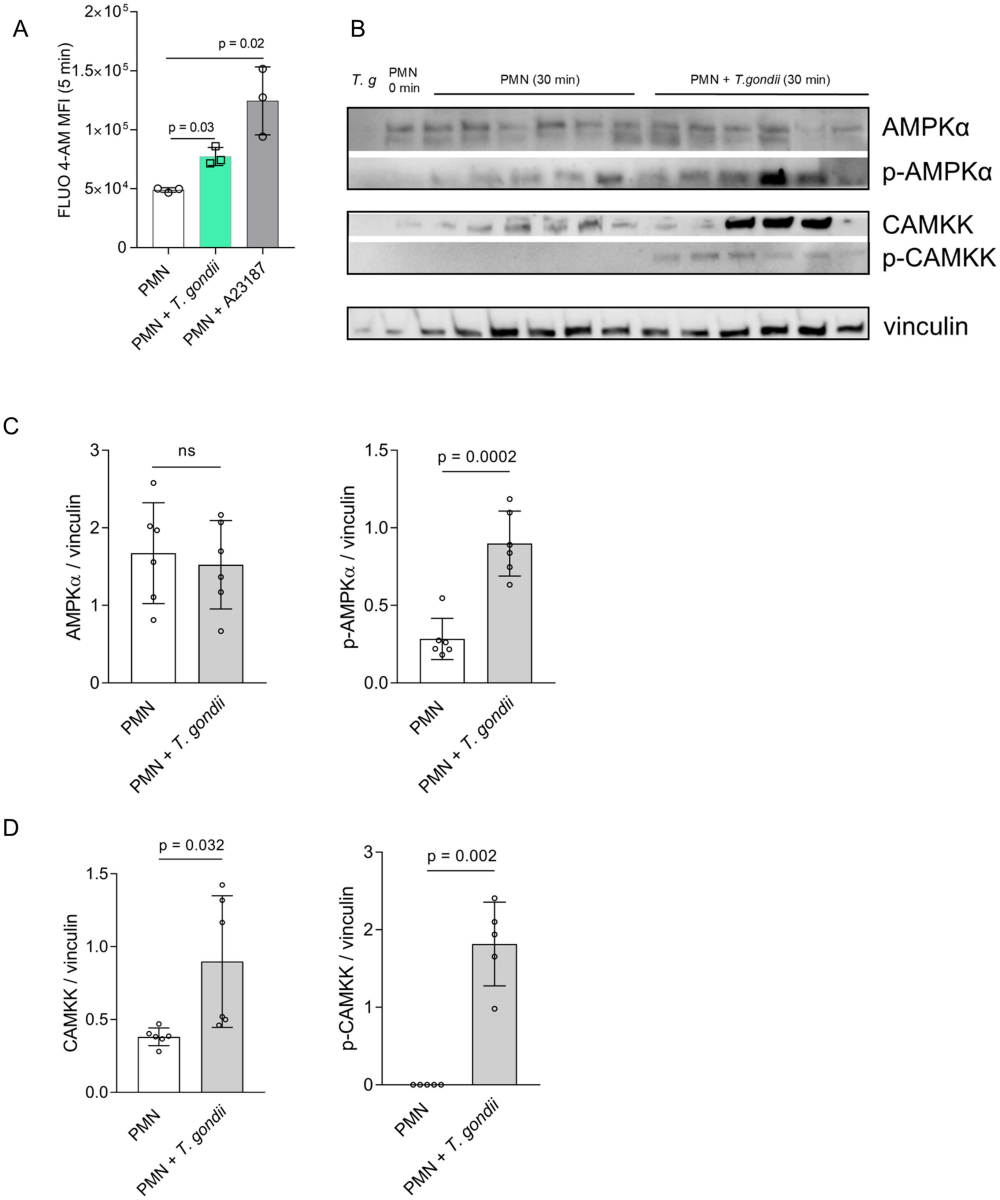
*Toxoplasma gondii* exposure induces cytoplasmatic calcium increase, AMPK and CAMKK phosphorylation in bovine PMN. Fluo-4 AM-loaded bovine PMN **(A)** (*n* = 3) were confronted with *T. gondii* or stimulated with the calcium ionophore A23187 for positive control. Fluo 4-AM derived fluorescence was measured by a flow cytometer and the mean of the fluorescence intensity after 5 min of co-incubation was represented as bar graph **(A)**. Bovine PMN isolated from peripheral blood from 6 different animals (*n* = 6) were exposed to *T. gondii* at 1:4 PMN: *T. gondii* ratio. After 30 min of co-incubation, protein extracts were generated from PMN and tested for AMPK, p-AMPK, CAMKK and p-CAMKK expression by Western blotting. The expression of vinculin was used as internal reference protein. **(B)** Representative Western blot and **(C)** densitometric analysis of protein bands for AMPK, p-AMPK. **(D)** Densitometric analysis for CAMKK and p-CAMKK. Bars in the graph represent the mean ± SD. *p* values were calculated by applying a Mann–Whitney test.

After confrontation of PMN with *T. gondii*, a rapid increase in neutrophil intracellular calcium concentration [Ca^2+^]_i_ was observed, being represented by fluorescence changes after 5 min of co-incubation (Figure 1A). On a statistical level, this reaction proved significant when compared to negative controls (Figure 1A). As expected, stimulation of bovine PMN with the calcium ionophore A23187 (5 μM) caused a stronger and sustained increase in [Ca^2+^]_i_ (Figure 1A), thereby proving as reliable positive control in the bovine system.

### 3.2 PMN exposed to *Toxoplasma gondii* show an increased AMPK and CAMKK phosphorylation

AMPK phosphorylation was studied at 30 min of PMN-parasite-interaction in *T. gondii*-exposed PMN (6 biological replicates) by WB-based analyses of PMN protein extracts (Figure 1B). A high heterogeneity between the biological replicates was observed, and the overall effect was that pAMPK, but not total AMPK, revealed a significantly enhanced expression after 30 min of co-culture (Figure 1C).

Given that cellular AMPK activity is regulated upstream by CAMKK (besides other regulators and signaling pathways), we also evaluated the expression and phosphorylation status of CAMKK at 30 min of PMN exposure to *T. gondii* (Figure 1B). Densitometric analysis of respective protein bands in WB (Figure 1D) indicated that both phosphorylated and non-phosphorylated CAMKK was upregulated in *T. gondii*-confronted PMN at 30 min of co-incubation, thereby indicating a sustained activation of CAMKK.

### 3.3 Analyses of autophagy-related proteins in *Toxoplasma gondii*-exposed PMN

**Figure 2:**
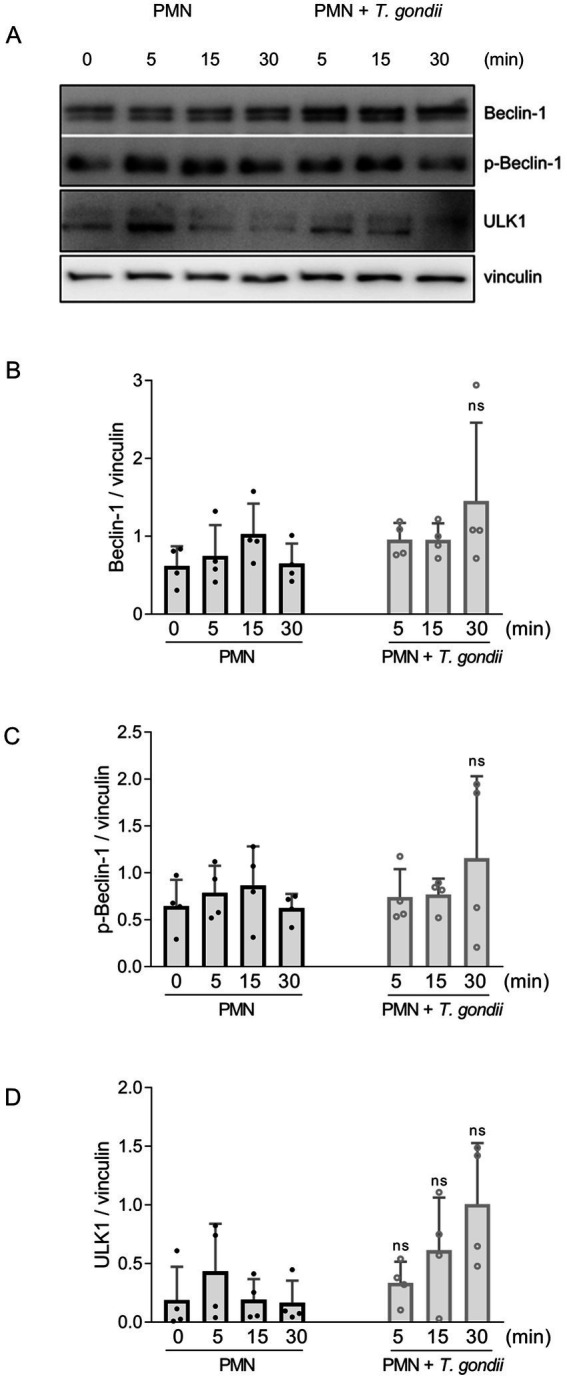
Studies on autophagy-related proteins on *T. gondii*-exposed PMN. Bovine PMN isolated from peripheral blood from four different animals (*n* = 4) were exposed to *T. gondii* at 1:4 PMN: *T. gondii* ratio. After 0–30 min of incubation, total protein extracts were generated from PMN and tested for Beclin-1, p-Beclin-1 and ULK1 expression by Western blotting. The expression of vinculin was used as internal reference protein. **(A)** Representative Western blot and densitometric analysis of protein bands for Beclin-1 **(B)**, p-Beclin-1 **(C,D)** ULK1 at 0, 5, 15 and 30 min of co-incubation. Bars in the graph represents mean ± SD. *p* values were calculated by unpaired two-tailed t-tests comparing control PMN vs. PMN incubated with *T. gondii* at each time point.

Since PMN exposure to *T. gondii* induced AMPK expression in bovine PMN, we also studied kinetics of early autophagic processes induced by *T. gondii* by evaluating the expression of Beclin-1 (in the phosphorylated and unphosphorylated form), and of ULK-1 at 5, 15, and 30 min of bovine PMN-*T. gondii* co-cultures (Figure 2A). The early expression profiles of these autophagy-related proteins showed an upregulation trend for ULK-1 after 30 min of co-incubation. However, after the densitometric analyses, none of the studied proteins showed a statistically significant increase in protein expression (Figures 2A–D).

### 3.4 Pharmacological AMPK activation enhances *Toxoplasma gondii*-induced NET formation

**Figure 3:**
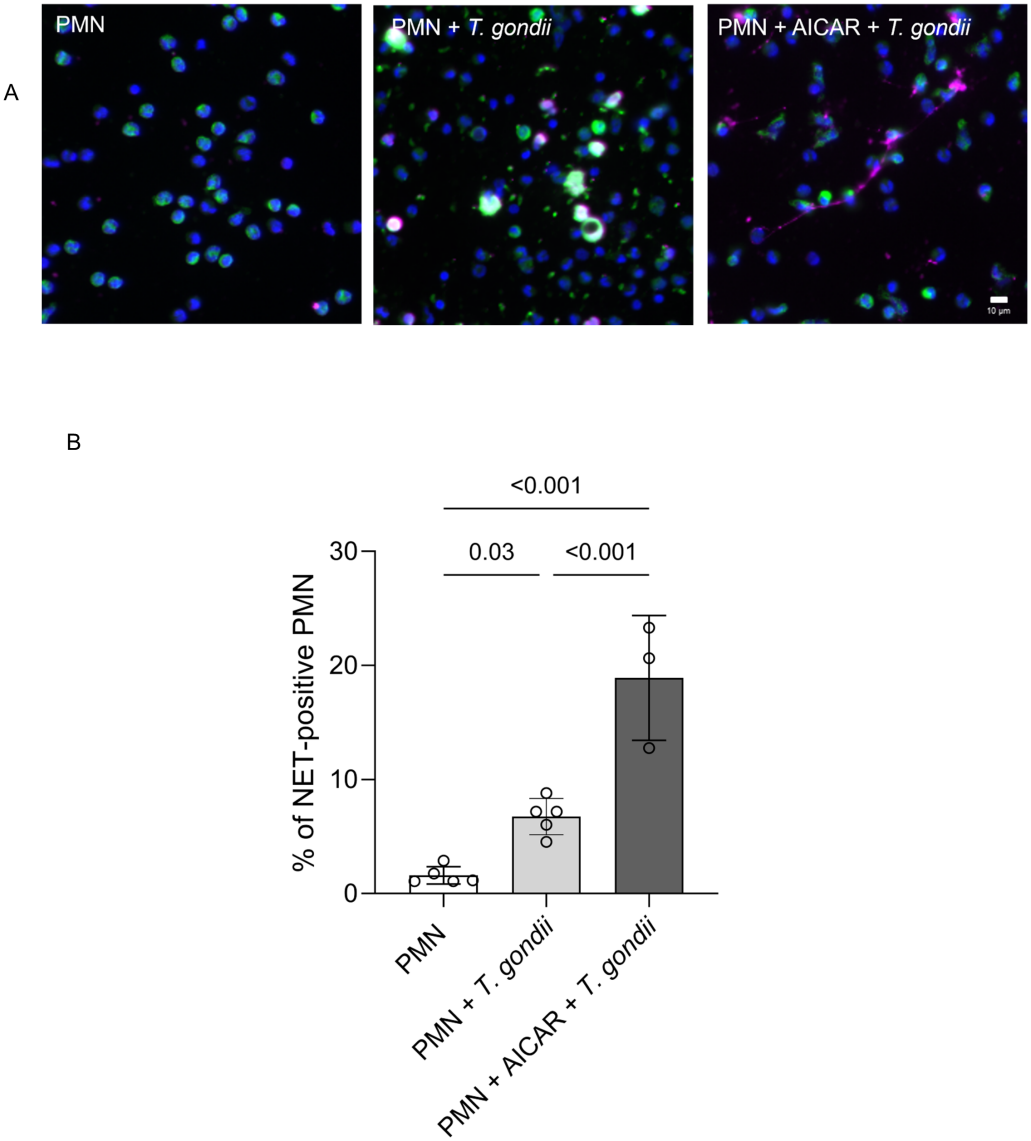
AICAR treatments enhance *T. gondii*-induced NET formation in bovine PMN. **(A)** Immunofluorescence images showing DNA (blue, DAPI), neutrophil elastase (NE, green) and DNA-histone complexes (magenta) in PMN (negative control), PMN-*T. gondii* -cocultures and AICAR-pretreated bovine PMN (30 min before *T. gondii* exposure). **(B)** The percentage of NET-releasing PMN was calculated by a semi-automated quantification method via image analysis (Image J, Fiji version) and is represented as bar graph, mean ± SD. *p*-values were calculated using a ANOVA test followed by a Tukey multiple comparison test.

To further confirm the role of AMPK in the process of early NETosis, we assessed potential additive effects of PMN AICAR pre-treatments on *T. gondii*--triggered NET formation (Figure 3). Therefore, bovine PMN were either directly exposed to *T. gondii* or additionally pre-treated with AICAR. Thereafter, NET formation was evaluated by immunofluorescence microscopy detecting DNA (blue, DAPI), NE (green) and DNA-histone complexes (magenta). Posterior image analyses used a semi-automatic method for NET quantification (Figure 3) (48). Current data showed that AICAR treatments had an additive effect on *T. gondii*-induced NETosis since *T. gondii* -driven NET formation was significantly enhanced by AICAR supplementation when compared to *T. gondii* alone exposure (Figure 3B) and to non-exposed bovine PMN.

### 3.5 *Toxoplasma gondii*-induced DNA release depends on MAPK- and SOCE-related pathways

**Figure 4:**
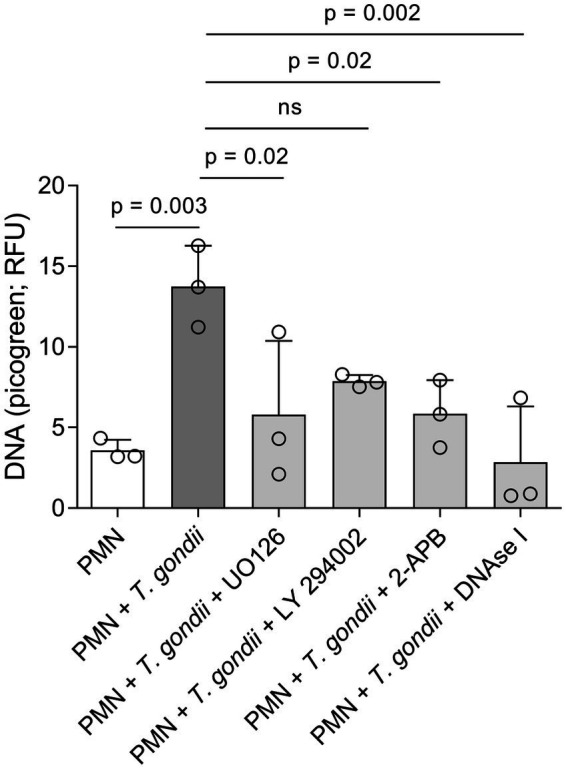
*Toxoplasma gondii*-induced DNA release in bovine PMN is dependent on ERK and SOCE signalling. Bovine PMN (*n* = 3) were pre-treated for 30 min with UO126 (50 μM), LY294002 (1 μM) or 2-APB (50 μM) before the addition of *T. gondii* (1:4 PMN: tachyzoites ratio). After 4 h of co-incubation, the Picogreen-derived fluorescence, corresponding to extracellular DNA amount, was determined in a plate reader. DNAse I (90 U) was added after the 4 h of incubation in the corresponding experiments to confirm the DNA-nature of the emitted fluorescence. Bars represent the mean ± SD. *p*-values were calculated by applying an ANOVA test followed by a Dunnet multiple comparison test.

To further expand and complement the results on the AMPK pathway, we also evaluated the effects of chemical inhibitors of the MAPK- (UO126), PI3K- (LY294002) and SOCE- (2-APB) related pathways. Current data confirmed that *T. gondii* exposure indeed induces DNA release from bovine PMN. This DNA release is dependent on both, the MAPK pathway and SOCE (Figure 4). DNA release as the main component of *T. gondii*-induced NETs was confirmed by DNAse I treatments that significantly diminished extracellular DNA counts (Figure 4; PMN + *T. gondii* vs. PMN + *T. gondii* + DNAse I).

## 4 Discussion

The NETotic process includes at least three well-defined phases. One of the major NETosis-driving forces is the entropic swelling of chromatin and the intracellular ATP reserve consumption. Here, we presented new data for the bovine system on early events of PMN exposure to *T. gondii* (within the time frame of 30 min) on the level of intracellular calcium concentration ([Ca^2+^]_i_), AMPK, CAMKK activation and the autophagic process.

[Ca^2+^]_i_ controls several bovine PMN functions like ROS production, degranulation and NETosis (36, 49). In the current study, exposure of bovine PMN to *T. gondii* indeed induced a rapid increase in [Ca^2+^]_I_ in PMN, which was later accompanied by an activation of both CAMKK and AMPK, with the former being commonly reported as Ca^2+^-dependent. Hence, CAMKK is able to activate AMPK in response to a rise in [Ca^2+^]_i_, independent of the AMP/ATP ratio (50). One of the underlying main mechanisms that mediates a transient, fast rise in [Ca^2+^]_i_ in PMN, is the so-called store-operated calcium entry (SOCE). SOCE, in turn, is well-documented to control ROS production, chemotaxis, degranulation and NET formation (36, 51, 52). The blockage of *T. gondii* tachyzoite-induced DNA release by the SOCE inhibitor 2-APB is in line with former data on parasite-driven NET formation using stages of the related parasites *E. bovis* (36) and *C. parvum* (53), thereby indicating a conserved role of SOCE in protozoa-induced NETosis. One of the targets of free cytosolic Ca^2+^ is the CAMK family of enzymes. In PMN, CAMK activities have been associated with PMN development and maturation (54), superoxide production (55), phagocytosis, migration, and adhesion. CAMKK is activated by IL-8 (56), fMLP and platelet activating factor (PAF) but not by phorbol 12-myristate-13-acetate (PMA) (57) in PMN and regulates functions via an ERK-MAP kinase-dependent mechanism (56). Previously, we already reported the activation of the CAMKK and AMPK pathways in bovine PMN being confronted with tachyzoites of the *T. gondii*-closely related parasite *B. besnoiti* (37, 38). Hence, thus current data confirms this observation and expands the proposed mechanisms to *T. gondii*-activated bovine PMN.

AMPK is a metabolic master regulator in eukaryotes. Besides its metabolic activity, in PMN AMPK induction enhances chemotaxis, bacterial killing, phagocytosis and MMP-8 secretion (39, 58). In the bovine system, PMN stimulated by agonists of hydroxycarboxylic acid receptor 2 (HCA2) and *β*-hydroxybutyrate showed an increased AMPK activity (59). Moreover, AMPK activity induces autophagy-related proteins like LC3, ATG5 and Beclin-1 in a low glucose (2.5 mM) setting (60). Mechanistically, AMPK promotes autophagy by directly activating ULK-1 during autophagosome formation (61). In agreement, in the current study *T. gondii* -exposed PMN showed enhanced AMPK phosphorylation after 30 min co-incubation, thereby correlating with an increase in autophagy-related proteins, such as ULK-1. Indeed, autophagy and NET formation have been demonstrated to be intrinsically linked, most probably due to some overlapping at protein level (38, 43, 45). In principle, current results in the context of previous studies (37), indicate that autophagy-related activation is stronger in *B. besnoiti*-confronted than in *T. gondii*-confronted bovine PMN. However, it remains currently entirely unclear, which specific parasite-derived factors may trigger this differences on autophagy-dependent mechanisms.

Pharmacological activation of AMPK in PMN by AICAR treatments results in cytoskeletal rearrangement and leading edge formation (39). Recently, we have shown that plain AICAR treatments result in PMN activation in the bovine system by significantly upregulating both, i. e. oxygen consumption rates (OCR) and extracellular acidification rates (ECAR) in bovine PMN exposed to *B. besnoiti* (37). In the current study, PMN treatments of *T. gondii* -exposed PMN with AICAR resulted in additive effects in case of NET formation. This finding is coherent with similar observations on *B. besnoiti*-confronted bovine PMN (37). In contrast to above mentioned findings, AICAR treatments led to diminished ROS production in PMA-activated human PMN, suggesting an overall stimulus-dependent response. In the human system, *T. gondii* -induced NET formation showed to be ROS- and glycolysis-dependent with the participation of gasdermin D and NE (20). Interestingly, the percentage of PMN performing NETosis in response to encounter in human neutrophils is higher (approximately 20%) (20) than in bovine ones, as presented here (10%), indicating a possible host species-specific effect, besides the potential impact of differential experimental settings. Referring to the here stated inhibition of parasite-driven extracellular DNA release by treatments with both the SOCE inhibitor 2-APB and the MAPK pathway inhibitor UO126, current findings are in line with observations on bovine PMN confronted with other coccidian stages, such as *E. bovis* sporozoites and *N. caninum* tachyzoites (36, 62). The observed lack of effect on the LY294002 (PI3K inhibitor) treatment is probably due to the small sample size and thus more biological replicates or microscopic analyses of NET formation are necessary to propose more precise conclusions. Altogether, these results suggests a conserved nature of these canonical activation pathways in bovine PMN driven by encounter with different apicomplexan parasites species and stages. Notably, another protozoan but non-related parasite, *Leishmania donovani*, induces autophagy in human PMN, depending on ROS production, AMPK activation and PI3K/Akt and ERK/MAPK signaling pathways (35). The authors were able to demonstrate that the augmented autophagy was a prerequisite for later macrophage-mediated uptake of infected PMN, thus promoting *L. donovani* infection (35, 63).

Several methods exist in the literature describing how NET formation can be studied or quantified. Immunodetection of NET markers as NE and MPO on the NET-forming chromatin fibers is recommended as the first choice for NET visualization and quantification (64). Automatic microscopic analyses for NET quantification developed for human and mouse PMN cannot be directly transferred to other systems as the equine and bovine without adjustments due to specie-specific differences in cellular and nuclear morphology (65, 66). On the other hand, techniques detecting free DNA as the main component of NETs are fast and cost effective in screening the effect of molecules on NET formation in virtually all species, but are less sensitive than microscopic analysis (64). The main drawback, however, is that DNA detected by probes as picogreen or Sytox orange are not able to discriminate between NET-DNA, DNA derived from necrosis or pathogen-derived DNA and thus the results must be interpreted with caution (65).

Altogether, current findings highlight the complex interplay between protozoan parasites and host-dependent innate immune responses. Overall, current data are consistent with the hypothesis that *T. gondii* encounter activates bovine PMN via a CAMKK-/AMPK−/ /NETosis-dependent mechanism.

## Data Availability

The raw data supporting the conclusions of this article will be made available by the authors, without undue reservation.
